# Amino acid-specific isotopes reveal changing five-dimensional niche segregation in Pacific seabirds over 50 years

**DOI:** 10.1038/s41598-024-57339-w

**Published:** 2024-04-03

**Authors:** Francis van Oordt, Antonio Cuba, Emily S. Choy, John E. Elliott, Kyle H. Elliott

**Affiliations:** 1 Department of Natural Resources Sciences, McGill University, Sainte-Anne-de-Bellevue, QC Canada; 2 Instituto del Mar del Perú, Callao, Peru; 3 Biology Department, McMaster University, Hamilton, ON Canada; 4 Science & Technology Branch, Environment and Climate Change Canada, Delta, Canada

**Keywords:** Stable isotope analysis, Ecological modelling, Food webs

## Abstract

Hutchison’s niche theory suggests that coexisting competing species occupy non-overlapping hypervolumes, which are theoretical spaces encompassing more than three dimensions, within an n-dimensional space. The analysis of multiple stable isotopes can be used to test these ideas where each isotope can be considered a dimension of niche space. These hypervolumes may change over time in response to variation in behaviour or habitat, within or among species, consequently changing the niche space itself. Here, we use isotopic values of carbon and nitrogen of ten amino acids, as well as sulphur isotopic values, to produce multi-isotope models to examine niche segregation among an assemblage of five coexisting seabird species (ancient murrelet *Synthliboramphus antiquus*, double-crested cormorant *Phalacrocorax auritus*, Leach’s storm-petrel *Oceanodrama leucorhoa*, rhinoceros auklet *Cerorhinca monocerata*, pelagic cormorant *Phalacrocorax pelagicus*) that inhabit coastal British Columbia. When only one or two isotope dimensions were considered, the five species overlapped considerably, but segregation increased in more dimensions, but often in complex ways. Thus, each of the five species occupied their own isotopic hypervolume (niche), but that became apparent only when factoring the increased information from sulphur and amino acid specific isotope values, rather than just relying on proxies of *δ*^15^N and *δ*^13^C alone. For cormorants, there was reduction of niche size for both species consistent with a decline in their dominant prey, Pacific herring *Clupea pallasii,* from 1970 to 2006. Consistent with niche theory, cormorant species showed segregation across time, with the double-crested demonstrating a marked change in diet in response to prey shifts in a higher dimensional space. In brief, incorporating multiple isotopes (sulfur, PC1 of *δ*^15^N [baselines], PC2 of *δ*^15^N [trophic position], PC1 and PC2 of *δ*^13^C) metrics allowed us to infer changes and differences in food web topology that were not apparent from classic carbon–nitrogen biplots.

## Introduction

Ecological niches are n-dimensional spaces or hypervolumes that describe the position of species by a complex suite of variables, both physical and biological^1^. The dimensions in an n-dimensional hypervolume usually include environmental factors that affect organismal performance, physiological limits, or morphological traits, as well as food resources or specific habitat needs, and can partly reflect important ecological patterns. N-dimensional niches have now been widely used to describe biological systems^2,3^ and explain functional diversity^4^, species morphological differences and taxonomy^5^, among other ecological variables. Often the aim of constructing communities’ niches is to detect patterns such as ecological segregation among species or groups within species (e.g. age classes or sexes)^6,7^, which is critical to modelling niche shifts caused by anthropogenic factors including habitat loss, pollution and particularly climate change^8,9^. Such segregation is expected to be the ‘ghost’ of past competition such that species currently exploit resources or have minimized competition under most circumstances^10–12^. For example, some sympatric seabird species segregate their foraging space or prey at several geographic and temporal scales^13–16^.

The principle of competitive exclusion states that complete competitors (with completely overlapping niches) cannot coexist^17–19^. Niche segregation, derived from differentiation, leading to coexistence among species and within species has been observed in a range of taxa, from plankton to songbirds^20–23^. If we consider a species’ hypervolume within Hutchinson’s n-dimensional niche space, then niche segregation implies that there is limited overlap of that hypervolume with coexisting species^7^. Seabirds are intriguing in this regard because they often include large populations of several species with apparently overlapping habitats, diets and foraging areas^24,25^. However, recent tools have shed light on how seabirds partition their foraging habitat, diet, and other components of their niche hypervolume^15,26^.

In predators that show high inter- and intra-specific competition dietary partitioning is an important component of niche diversification^27^. The study of trophodynamics, within marine communities allow us to understand changes in time and space of such niche diversification and trophic relationships^28^. Direct diet sampling can be invasive (i.e. killing animals to sample stomachs) or be biased due to differential digestion of prey items, while behavioural observations can also be biased and difficult for marine species that forage over large areas offshore^29^. Stable isotope values of carbon (*δ*^13^C) and nitrogen (*δ*^15^N), representing the relative proportion of ^13^C/^12^C and ^15^N/^14^N in tissues, have proven to be reliable indicators of diet, and consequently can be used to delineate trophic assemblages in marine ecosystems^28,30–32^. In particular, *δ*^13^C can be used to infer feeding habitat (such as pelagic or benthic/coastal), whereas *δ*^15^N is commonly used to provide an index of trophic position^33^. However, both bulk isotopic values are unable to reflect the influence of baseline values of the ecosystem, therefore careful interpretation if often needed^34^. Layman metrics can be used to describe trophic levels (range of *δ*^15^N), niche diversification (*δ*^13^C range), overall density of species packing (mean of the Euclidean distances to each species’ nearest neighbor in bi-plot space), among other indices that help describe trophic niches and relationships^35^. The use of these metrics in characterizing trophic dynamics can help detect different interactions within the community such as competition (by increased trophic redundancy) or high predation levels (increase in trophic diversity)^36^.

However, most studies of niche partitioning using stable isotopes have only employed *δ*^13^C–*δ*^15^N biplots^35,37,38^. In some cases, “*δ*-spaces” can be converted into “p-spaces” using mixing models to quantitively assign diet proportions^32^. However, two variables are unlikely to be able to resolve complex food webs^39^. The use of additional isotopes provides additional degrees of freedom especially when trying to identify prey sources with the use of the above-mentioned mixing models. The addition of a third dimension in community ecology analysis can provide important information overlooked with only two isotopic dimensions^40^. Three -dimensional approaches to look at isotopic niche segregation, such as incorporating sulphur (*δ*^34^S), have proven to be efficient in detecting segregation in marine species^41^. This has been reported also in seabirds when marine epipelagic species are more enriched in *δ*^34^S compared to benthic or coastal species, proving to good indicator independent of trophic level (unlike bulk carbon isotopes)^42^. The use of compound specific isotopic analysis, such as using carbon and nitrogen isotopes of amino acids, overcomes some biases present in bulk analysis of *δ*^13^C and *δ*^15^N^43,44^. Moreover, current tools to look at n-dimensional niches are increasing in the literature, including re-sampling and Bayesian methods to produce metrics for isotopic niches^45^. Incorporating amino acid specific isotopic values into ecological studies provides new levels of information that allows to differentiate trophic and baseline signals in the trophic webs that would otherwise be masked in bulk isotopic values^43,46^. The use of trophic indicators such as nitrogen isotopic values of glutamic acid (Glx) may allow to discriminate baseline shifts and also improve predictions trophic position. Such tools allow for testing of hypotheses about how niches vary across dimensions (e.g. changes in overlap and segregation patterns, more distant centroids, smaller volumes).

Niches change over time due to natural and anthropogenic changes. In particular, niche segregation may only be apparent when resources are limited; during resource pulses, all species may be able to take advantage of the same abundant food sources^47,48^. In marine habitats, overfishing^49^, marine pollution^50^, and other stressors^51^ could cause variation in isotopic niches that could reflect important changes in species populations. Stable isotopes allow tracking of diet over long time scales due to archived specimens and may provide insights on important trophic changes^52^. These changes may occur over very long periods, hundreds of years or decades^49,53^, or shorter periods, such as seasons or consecutive years^30,54,55^. Thus, isotope measurements in historical samples link population trends with diet shifts across time. Nonetheless, most studies examine only one (*δ*^14^N) or two (*δ*^13^C and *δ*^15^N) isotopes, which may mask niche variation occurring in other dimensions. Therefore, producing clear niche metrics and understanding fine patterns of niche shifts in seabird assemblages through time may allow for better management and conservation of species due to these current threats (e.g. increasing pollutant loads in oceans, climate change, etc).

Here, we incorporate an n-dimensional approach, comparing 1, 2, 3, and 5-dimension models, to build isotopic niches with *δ*^13^C and *δ*^15^N of 10 amino acids and bulk δ^34^S in an assemblage of five coexisting seabirds (ancient murrelet, double-crested cormorant, Leach’s storm-petrel, rhinoceros auklet, pelagic cormorant) that breed along the southern coast of British Columbia. We test the idea that seabirds overlapping in *δ*^13^C and *δ*^15^N biplots will differ in segregation patterns when using multiple stable isotopes in higher dimensions (2, 3, and 5 dimensions). Especially, trophic relationships will be more apparent when using amino acid specific nitrogen values as indicators, e.g. reflecting higher trophic level for cormorants, compared to storm-petrels or auklets. Moreover, we expect that in higher dimensions, most Layman community metrics should differ as all five species may change patterns of segregation from each other, but relative niche volume among species should remain constant. As the prey base of cormorants changed over time, we predicted that niche centroids would vary, and overlap would decrease, but overall niche size would remain constant in higher dimensions.

## Methods

### Dataset and study species

We studied five species (ancient murrelet, double-crested cormorant, Leach’s storm-petrel, rhinoceros auklet, pelagic cormorant) using whole eggs collected since 1969 and archived at the National Specimen Bank (National Wildlife Research Centre [NWRC], Ottawa, Ontario) as part of the long-term contaminants monitoring program initiated in 1968 by the Canadian Wildlife Service (see Table S1 for final egg sample numbers per species). Seabird eggs are commonly used as a relatively non-invasive sampling source to represent the contaminant and dietery composition in the female adult prior to and during the egg-laying period. Double-crested cormorants *Phalacrocorax auritus* are generalist seabirds, inhabiting coastal nearshore to inland aquatic environments across North America^56^. They feed on diverse benthic and mid-water schools of fish in the British Columbia coast. Pelagic cormorants *Phalacrocorax pelagicus* forage mainly in deeper water on the continental shelf during the breeding season^57–59^. Rhinoceros auklets *Cerorhinca monocerata* (hereafter auklets) inhabit temperate waters of the northern Pacific^60,61^ and feed on epipelagic fish^62^. Ancient murrelets *Synthliboramphus antiquus* (hereafter murrelets) are offshore, sub-surface feeders and prey on zooplankton and small, schooling fish^63^. Leach's storm-petrels *Oceanodrama leucorhoa* (hereafter storm-petrels) are planktivorous surface feeders found in the northern Atlantic and Pacific Oceans^64^. While breeding, they feed closer to the colony on the continental shelf, whereas they feed hundreds of kilometres away when not breeding^64^. Eggs were sampled from islands and coastal sites on the Pacific coast of British Columbia, Canada: Cleland Island (storm petrels and auklets), Langara Island (murrelets), Lucy Island (auklets), Mandarte Island (cormorant), Mitlenatch Island (pelagic cormorants), Thomas Island (storm-petrels), and Thorton Island (storm-petrels) (Fig. S1). Maps were produced with the gpplot2^65^ and sf^66,67^ packages, and shapefiles from gadm.org. We followed the methods already presented in published contaminant research^42,68–70^. Sampling effort (frequency and sites sampled) varied since 1970 due to cost and logistics, and in some cases, eggs were collected during spring and early summer (late April to early July). Some egg samples were pooled into 1 g sub-samples from a total of 15, 5, or 3 samples after homogenization as described in work by Miller et al.^70^. Briefly, 1.5 g wet weight of aliquots were homogenized after removal from the eggshell and then subsampled into aliquots for preservation.

### Stable isotope analysis

Stable isotope analysis for bulk carbon, nitrogen, and sulphur was carried out using sub-samples of 1 mg freeze-dried eggs, loaded into tin, using a PDZ Europa ANCA-GSL elemental analyzer interfaced to a PDZ Europa 20–20 isotope ratio mass spectrometer (IRMS; Sercon Ltd., Cheshire, UK) at the Stable Isotope Facility at the University of California, Davis (http://stableisotopefacility.ucdavis.edu). Delta values were provided in parts per thousand (‰) and *δ*13C values have been lipid normalized^71^. Carbon and nitrogen stable isotopes of ten specific amino acids, both essential and non-essential (alanine, valine, glycine, isoleucine, leucine, proline, aspartic acid, phenylalanine, glutamic acid (Glx), lysine) were also analyzed at UC Davis Stable Isotope facility via GC-C-IRMS as described in Refs.^42,68–70^. Stable sulfur isotope we performed in a Europa Roboprep-20/20 EA-IRMS (lipids were not extracted from homogenates as lipid extraction is known to alter δ^34^S and lipids should not contain sulfur) as described by Elliott et al.^71^. Samples for specific amino acids were analyzed with an isotope cube elemental analyzer (Elementar, Germany) interfaced with a Finnigan DeltaPlus XP isotope ratio mass spectrometer (Thermo Germany) coupled with a ConFlo IV (Thermo Germany). Amino acids were liberated via acid hydrolysis and derived by methyl chloroformate. Methoxycarbonyl amino acid methyl esters were then injected in splitless (^15^N) mode and separated on an Agilent DB-23 column (30 m × 0.25 mm ID, 0.25 μm film thickness). Once separated, the esters were converted to N^2^ in a combustion reactor at 1000 ◦C. Water was subsequently removed through a nafion dryer. During the final step of the analysis, N2 entered the IRMS. Pure reference N^2^ was used to calculate provisional δ- values of each sample peak. Next, isotopic values were adjusted to an internal standard (e.g. norleucine) of known isotopic composition. Final δ-values were obtained after adjusting the provisional values for changes in linearity and instrumental drift such that correct δ-values for laboratory standards were obtained. Laboratory standards were custom mixtures of commercially available amino acids that had been calibrated against IAEA-N1, IAEA-N2, IAEA-N3, USGS-40, and USGS-41. A final subset of 63 samples (Table S1) for five species of seabirds with a complete set of sulphur isotopes, and carbon and nitrogen amino acid isotopes was used for all analyses.

### Statistical methods

All data processing and analyses were performed using the R software^72^. Because studies have found less support for the Glx-Lys difference in high consumers^73^, we tried a different approach that was agnostic to the meaning of *δ*^15^N in amino acids by using all the variance of the isotopic values of each compound by means of principal component analysis (PCA). PCA were performed on the 63-sample subset for both sets of carbon and nitrogen amino acid-specific isotopic values, independently using the prcomp() function, with scaling, in base R. We used the first principal component (PC) of the nitrogen amino acids (PC1-Nss) for one-dimensional analyses, as *δ*^15^N is a classic basic indicator of trophic level segregation among sympatric predators^39^. We used the first PCs of carbon and nitrogen amino acids (PC1-Css and PC1-Nss) for the two-dimensional analyses. We included the standardized value of sulphur (referred to as *stdDeltaS*) to these previous two dimensions for the 3-dimensional approach and incorporated the second PC of both carbon and nitrogen amino acid stable isotope values for the 5-dimensional approach (see Table 1). PC1-Css and PC1-Nss showed a strong correlation with bulk independently measured isotope values of carbon and nitrogen^39^, and therefore PC1 values of amino acids were considered also as proxies of bulk carbon and nitrogen isotopes.

**Table 1:** Components incorporated in each dimensional approach.

Model approach	Dimensional components
1-dimensional	PC1 of *δ*^15^N in amino acids (PC1-Nss)
2-dimensional	PC1 of *δ*^13^C and PC1 of * δ*^15^N in amino acids (PC1-Css, PC1-Nss)
3-dimensional	PC1 of *δ*^13^C and PC1 of * δ*^15^N in amino acids, and standardized *δ*^36^ S (PC1-Css, PC1-Nss, stdDeltaS)
5-dimensional	PC1 and PC2 of *δ*^13^C in amino acids, PC1 and PC2 of *δ*^15^N in amino acids, and standardized *δ*^36^ S (PC1-Css, PC1-Nss, stdDeltaS, PC2-Css, PC2-Nss)

Principal components of amino acids are labelled with the corresponding isotope for carbon and nitrogen (e.g. PC1-Css for carbon isotope values in amino acids, or PC1-Nss for nitrogen isotope values in amino acids).

The dimensions produced from the PCA analyses proved to be a good proxy for isotopic values of carbon and nitrogen. Scores of PC1 of carbon (PC1-Css) were equally loaded in all amino acids (ranging from 6.16 to 12.32%) and strongly correlated with bulk carbon values (r = − 0.88) (Table S2). PC2-Css had the highest loading for proline (50.18%), a non-essential amino acid. PC1 of Nitrogen (PC1-Nss) was mainly loaded on alanine, isoleucine, leucine, and valine (14.96 to 16.32%), the first three being trophic amino acids, and highly correlated with bulk nitrogen isotope values (r = 0.57). PC2-Nss was loaded on aspartame, and phenylalanine (23.67 and 30.57 respectively), trophic and source amino acids, respectively. Additionally, to confirm our approach, we contrasted the first two PCA components of the *δ*^15^N values in amino acids with several trophic indices (Glx-Lys, Glx-Phe, and two trophic position indexes for single and mean amino acid values^74–76^), and found strong correlations with either PC2 (r >|0.6| in all cases, see Fig. S2). We could assume that PC1-Nss was an indicator of baseline nitrogen values, and PC2-Nss closely reflected trophic level. Similarly, we checked for the the correlation of the PC1 raw carbon (uncorrected) values and the PC1 of corrected *δ* values of amino acids for Suess effect for the Gulf of Alaska region, using the SuessR package^77^, and found a very strong correlation (r = 0.998, also Fig. S2).

The package nicheROVER^45^ was used to model the distribution of the different niche isotopic components using a Bayesian inference framework, incorporating uncertainty into the analysis, and a method insensitive to sample size (therefore the increase of sample size will not cause random increases in niche region). The default ‘non-informative’ priors were used in nicheROVER. We sampled 100,000 posterior distributions to calculate centroid locations and probability percentiles for all species and overall community metrics (described below) to be used in comparing dimensional approaches. The nicheROVER package also produces estimations for niche size (described as the probability in n-dimensional space) and niche overlap (the 95% probability of one species falling into the niche of another). Overlap estimates incorporated 95% of the data.

To compare differences between independent dimension centroids and niche sizes between species, we calculated the Bhattacharyya Coefficient^78^. This coefficient estimates the probability of overlap between two posterior distributions. To identify if niches of each species had different positions in isotopic space the posterior estimates *µ* were divided into a null (*n*) and test (*t*) equally sized distributions^41^. We used those estimates to produce a conservative probability estimate that the centroid locations differ between species, by calculating the probability that the distance between two centroid values in the test distributions (*µ*_1t_ and *µ*_2t_) was greater than the distance between the test and null distributions for *µ*_1_ and *µ*_2_, respectively:

1 $$P\left[D\mu 1,\mu 2\right]>0 =\frac{\Sigma \left(D\left[{\mu }_{1},{\mu }_{2}\right]- D\left[{\mu }_{1t},{\mu }_{2t}\right]- D\left[\mu 2t,\mu 2n\right]>0\right)}{total \,\,number \,\,of\,\, posterior \,\,estimates\,\, in\,\, null\,\, or \,\,test\,\, distributions}.$$

We used the 100 000 draws from the posterior distribution extracted from the nicheROVER models, and calculated isotopic range for each tracer, representing the different mean of diversification in trophic level or niche, centroid distance (CD), representing species spread in space, nearest neighbour distance (NND), the density of species packing or niche redundancy, and standard deviation of the nearest neighbor distance (SDNND) following the methods of Layman et al.^35^, to assess at how increasing dimensionality may influence the representation of community structure.

A spider chart was used to plot independent centroid locations for each dimension and all species. Three-dimensional plots were produced with the mean values of sigma and mu (covariance and means posterior values) following Rossman et al.^41^. Boxplots of the probability distribution of CD, NND, SDNND, and ranges for all variables for the whole community in all dimensional approaches are presented in the Supplementary Materials.

We additionally evaluated the changes in cormorants (the two species with the largest sample sizes that allowed for such comparison) niches for 2D, 3D, and 5D dimensional approaches only between the periods of 1970–1989 and 1990–2006.

## Results

The use of stable isotopic values to describe ecological segregation in communities or assemblages is gaining momentum^36,79^. Moreover, ecological patterns and segregation often need more than just two indicators to describe observable differences and pattern shifts in time^40,41^. The use of Layman metrics in a high-dimensional approach to get a better approximation to the n-dimensional ecological niche, described by Hutchinson, should allow for better observation of ecological patterns. Of the Layman metrics analysed here, centroid locations of niches for each species and niche sizes were fairly consistent in all dimensional approaches, but overlap (and therefore segregation) patterns changed not only in proportion to dimensionality when comparing lower (1D and 2D) to higher dimensional approaches (3D and 5D) (Tables 2, 3; Figs. 2, 3).

**Table 2:** Niche sizes for five species of seabirds on the coast of British Columbia.

	1D	2D	3D	5D
Leach’s storm petrel	4.38 (2.94–6.75)	18.02 (12.67–33.23)	5.06 (3.37–10.68)	**33.03 (20–85.13)**
Ancient murrelet	7.62 (4.47–13.7)	20.24 (13.49–48.7)	5.72 (3.57–16.27)	1.37 (0.8–5.16)
Pelagic cormorant	5.21 (3.85–7.23)	27.81 (20.78–43.77)	19.68 (13.74–33.77)	137.11 (86.72–269.57)
Rhinoceros auklet	5.39 (3.55–8.52)	31.24 (21.64–59.73)	54.38 (34.78–117.28)	298.45 (170.84–771.4)
Double-crested cormorant	5.29 (3.83–7.47)	41.36 (30.09–66.11)	120.35 (81.87–211.32)	689.04 (425.32–1408.16)

**Figure 1:**
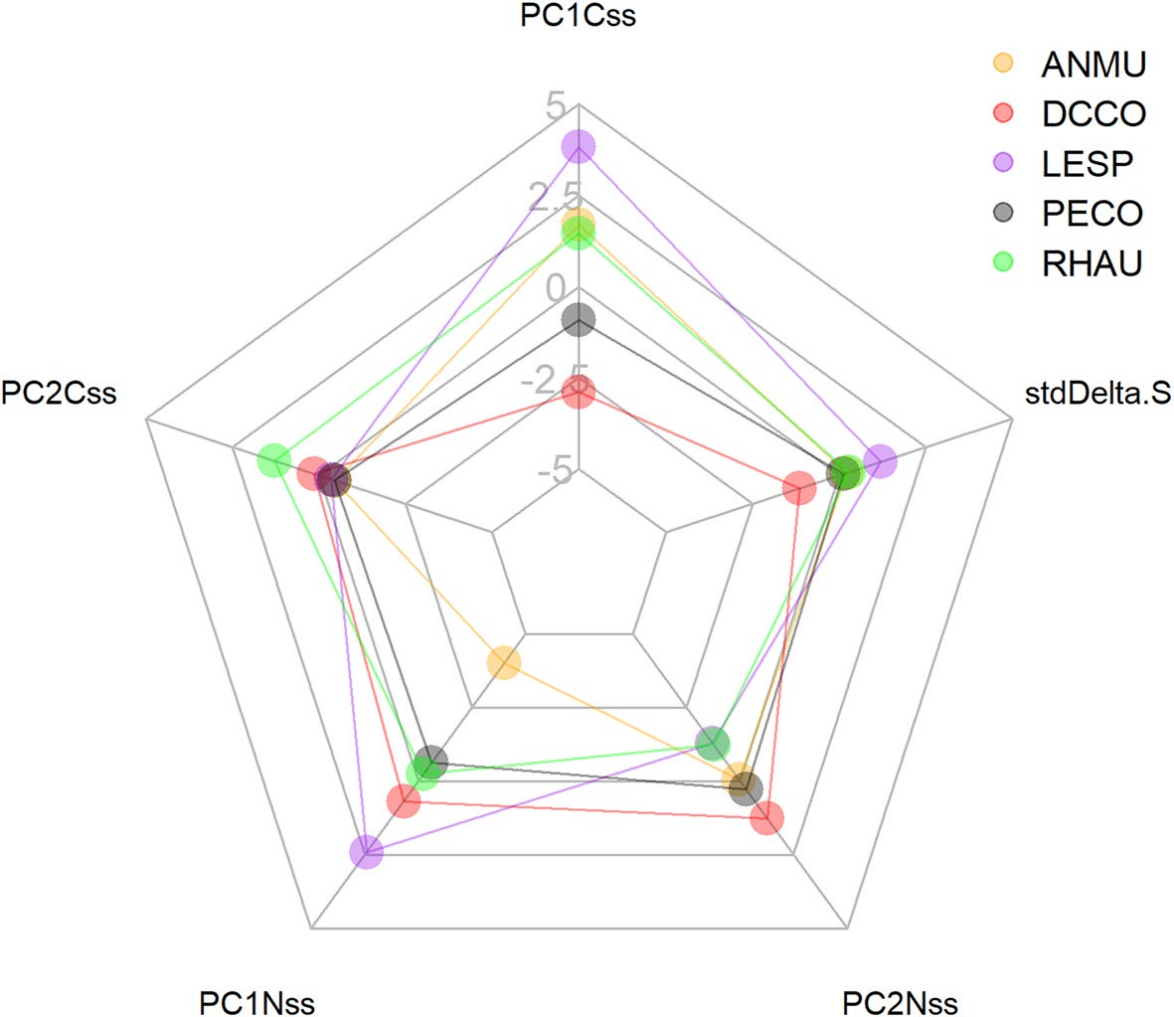
Average centroid locations for all species in 5 dimensions from 100 000 samples drawn from the posterior distribution (where PC1Css, PC2Css, PC1Nss, and PC2Nss are the first and second principal component of carbon and nitrogen isotope values of amino acids, respectively. stdDeltaS is the standardized value of sulphur values). *ANMU* ancient murrelet, *DCCO* double-crested cormorant, *LSPE* Leach’s storm-petrel, *PECO* pelagic cormorant, *RHAU* rhinoceros auklet. These are relative positions when comparing one species to another and polygons do not represent niche size or shape (the PCA axes were not rotated).

Mode and 5% and 95% quantiles of the posterior distribution from 100,000 samples are shown. Bolded values are those representing shifts in increasing trend size of niches into higher dimensional approaches. See Fig. 1 for species abbreviations.

**Table 3:** Centroid distance (CD), nearest-neighbor distance (NND), and standard deviation of the NND (SDNND) for all five species of seabirds in 1D, 2D, 3D, and 5D.

	CD	NND	SDNND
1D	1.63 (0.03–4.53)	0.003 (0–0.013)	0.004 (0.001–0.01)
2D	2.87 (0.5–4.85)	0.012 (0.002–0.042)	0.01 (0–0.032)
3D	2.95 (0.61–4.94)	0.055 (0.018–0.148)	0.03 (0.01–0.08)
5D	3.27 (1.43–5.05)	0.00002 (0–0.00006)	0.00004 (0–0.00011)

Mean and quantiles (2. 5% and 97.5%) of the posterior distribution from 100,000 samples.

### Species centroid locations in 2D, 3D, 5D

Species relative values for each dimension were preserved from 2 to 5D (Fig. 1, Table S3). Storm-petrels tended to be positioned the farthest from the rest of the species, whereas the two cormorant species, were consistently close to each other, but not always overlapping significantly (Table S4). The two alcids differed in only two axes, PC1-Css and sulphur (std-*δ*^36^S) (Bhattacharyya coefficient probability of 0.95 and 0.63), as expected from their different diets. Overall, no species coincided in all dimensions with another species, indicating community segregation.

All species showed different overall centroid locations. All species occupied a different location in the iso-space across all dimensional approaches (Table S5A–C). The difference between centroid locations was somewhat consistent among dimensions, with some interesting shifts that can be seen in Table S5D–F. For example, storm-petrels and double-crested cormorants were more distant to each other than any other pair of species in all dimension approaches. Conversely, both cormorants were the closest species in 2D and 5D; but not in 3D, where pelagic cormorants and auklets were the closest species to each other. Interestingly, we can see a clear shift in species in PC1Nss and PC2Nss, reflecting important differences in baseline and trophic signals. For example, storm-petrel had highest values of PC1-Nss (baseline), but lowest values of PC2-Nss (trophic position) together with the rhinoceros auklet, and double-crested cormorant had higher trophic level.

### Niche sizes in 1D, 2D, 3D, 5D

Niche size remained relatively constant from 2 to 5D (Table 2). Double-crested cormorants had the largest 2D niche, though similar in size to alcids (niche size 31.24 and 41.36, Bhatt coef. prob. = 0.89, Table S6, Fig. S2). By a statistically significant margin (> 0.8 probability), double-crested cormorants were found to have the largest niche size in higher dimensional approaches, followed by auklets, and pelagic cormorants. Storm-petrels had the smallest niche in 2D and 3D, but not in 1D or 5D, where murrelets had the smallest niche size. Niche sizes were very similar in lower dimensions, with almost half of the pairs of species having overlapping niche sizes with a probability greater than 80% (for niche size overlapping probabilities see Table S6). Niches sizes increased in higher dimensions, with only storm-petrels and murrelets having overlapping niche sizes in 3D. No pair of species had overlapping niche sizes in 5D (Tables 2 and S6).

### Niche overlap among species

**Figure 2:**
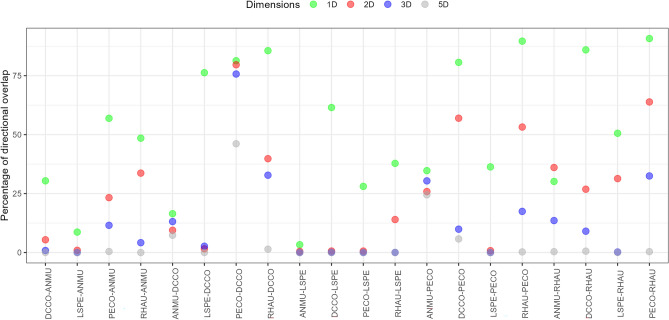
Graphical representation of the percentage of directional niche overlap between all pairs of species. Percentage of overlap represents first species overlapping on second species. See Fig. 1 caption for species abbreviations.

**Figure 3:**
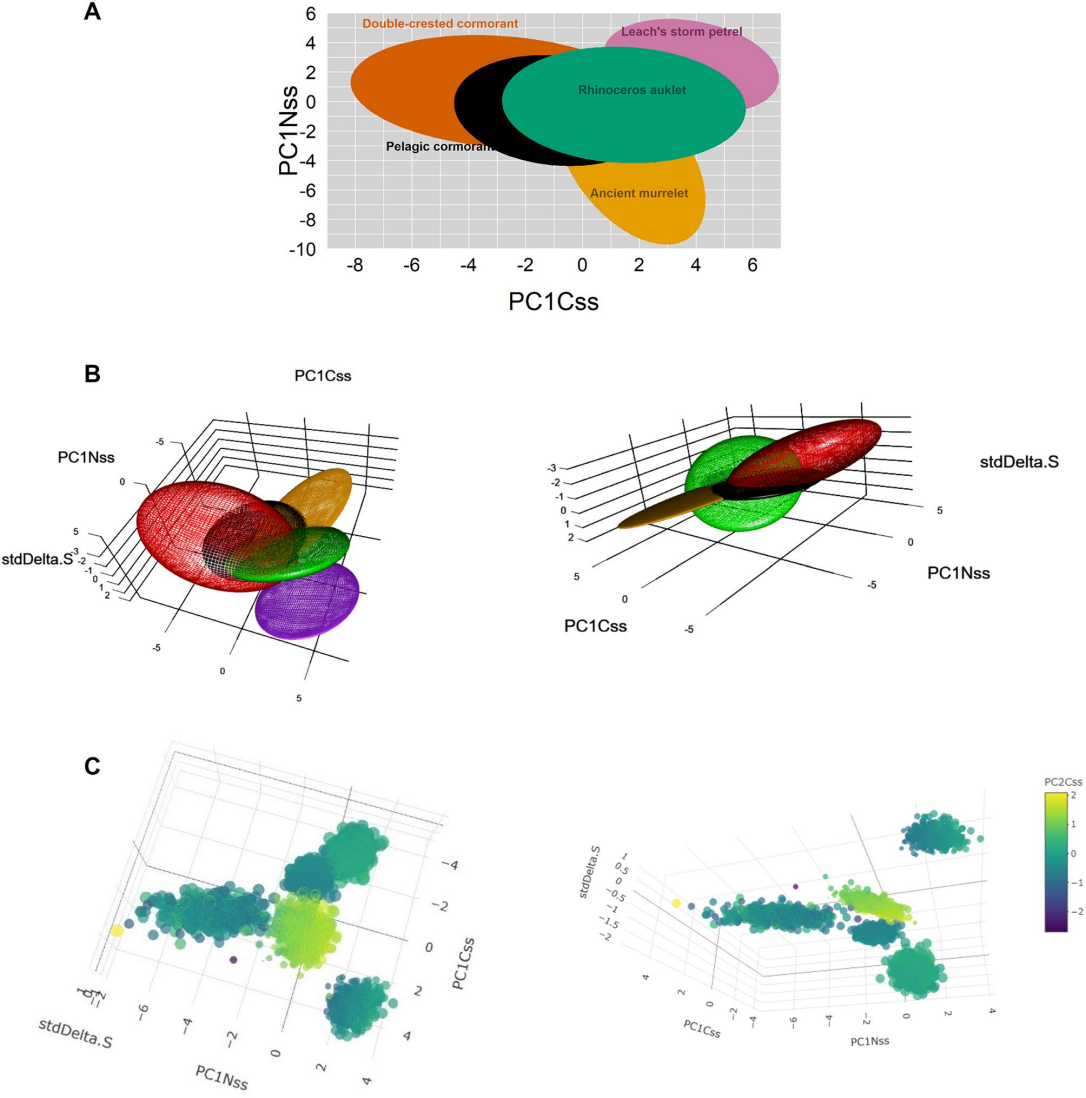
Graphical representation of niche sizes in (**A**) 2D (covariance ellipses), (**B**) 3D (covariance ellipsoids) [yellow = ancient murrelet, red = double-crested cormorant, purple = Leach’s storm-petrel, black = pelagic cormorant, green = rhinoceros auklet], and (**C**) 5D (simple five-dimensional plotting of 1 k random points) for five species of seabirds in the British Columbia coast, where colour is the 4th dimension (PC2Css), and circle size the 5th dimension (PC2Nss).

High overlap (greater than 50%) between species pairs (in lower dimensions, 1D, with one exception, and 2D) is consistently reduced with an increase in dimensions (Figs. 2, 3). The effect was not solely due to dimensionality, as the pattern changed with each incorporated dimension. Less significant overlap in some species pairs varied with dimensional approaches; some pairs of species increased overlap from 2D to 3D, but then decreased greatly in 5D (e.g. murrelets overlapping with double-crested cormorant) (Table S7 and Fig. 2), whereas in other species, the decrease was minimal (murrelets on pelagic cormorant). In general, 5D overlap was significantly reduced as expected, but especially in those species with smallest niches. Double-crested and pelagic cormorants showed the greatest overlap among all pairs of species consistently in all dimensional approaches (Fig. 2).

### Dispersion metrics in 2D, 3D, 5D

Species spread (Centroid Distance, CD) for all 5 seabird species showed a small increasing trend from lower to higher dimensions (10% or less) (Table 3). On the other hand, an increasing trend observed for NND values and the standard deviation of NND is higher in magnitude, reflecting a variation in density and evenness of species packing, respectively. Independent dimension ranges varied mainly for PC1-Css (6.7 [4.83–7.95]) and PC1-Nss (6.5 [3.35–8.75]), whereas the rest of the dimensions ranged in less than 3 units (Table S12).

### Temporal change and overlap in isotopic niches of cormorants: 2D, 3D, 5D

**Figure 4:**
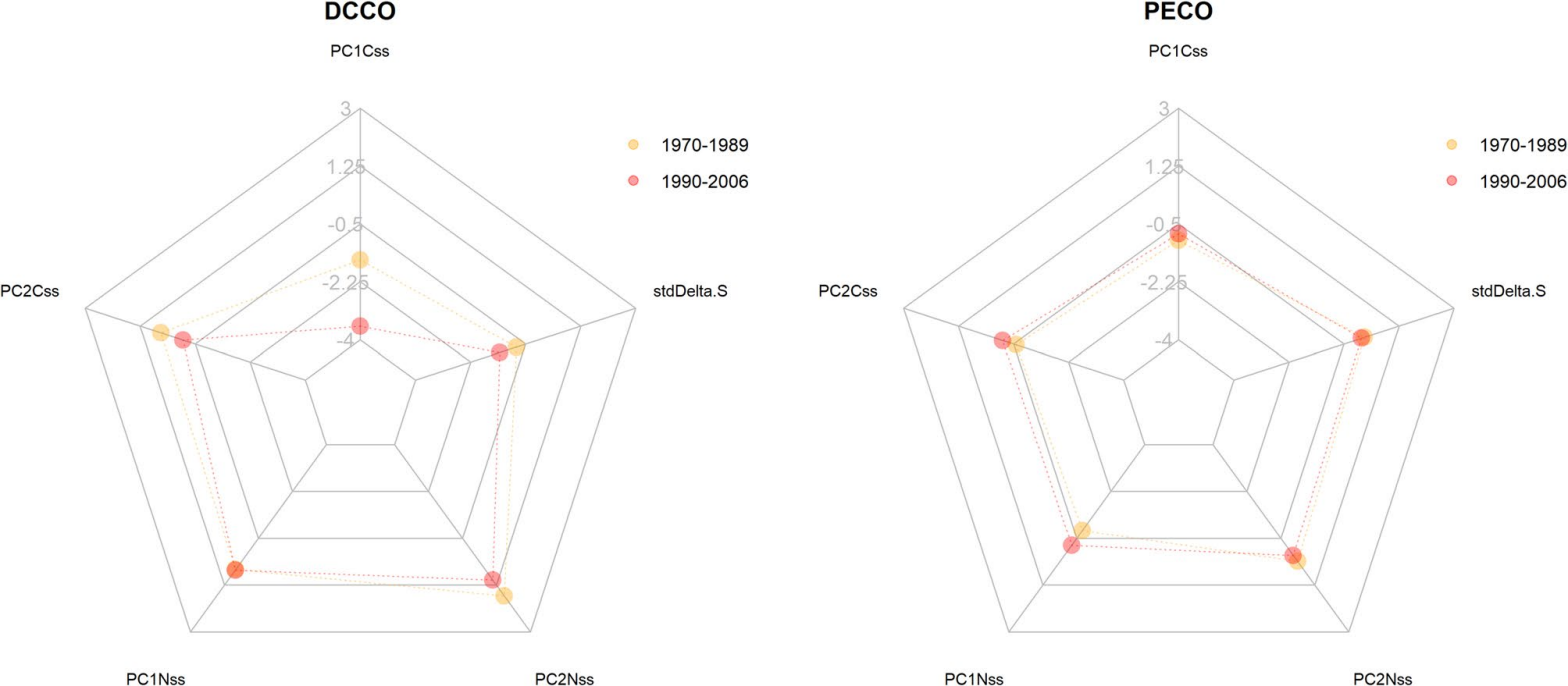
Centroid locations for DCCO and PECO in 5 dimensions in two time periods (where PC1Css, PC2Css, PC1Nss, and PC2Nss are the first and second principal components of carbon and nitrogen isotopes of amino acids, respectively. stdDeltaS is the standardized value of δ sulphur.

Niche sizes for both cormorant species decreased between the 1970–1989 and the 1990–2006 periods (Table 4). Pelagic cormorants decrease in niche size through time when more dimensions were incorporated into the model, whereas double-crested do not (Table 4). Double-crested cormorants showed shifts in most independent isotopic dimensions for all 5 axes, whereas pelagic cormorants had no important changes in any dimension (Fig. 4, Table S8). Double-crested cormorants showed a trend of niche change (distance between centroids of two time periods ≤ 2.4) in all three approaches, although the probability was low (≤ 0.6, Table S9), whereas pelagic cormorants showed a larger change (distance ≤ 0.87 and probability of ≤ 0.2). Conversely, both species showed little difference in overall centroid location (position in the iso-space) for their niches in different periods with double-crested cormorants having significant differences (above 50%) and a higher distance between periods, compared with pelagic cormorants (Table 5).

**Table 4:** Niche sizes for double-crested and pelagic cormorant during the 1970–1989 and 1990–2006 periods in the British Columbia coast.

		2d	3d	5d
Double-crested	1970–1989	68.757 (45.269–164.295)	69.467 (41.866–192.405)	93.679 (49.5–316.823)
	1990–2006	8.714 (6.164–16.208)	28.362 (18.259–58.255)	89.93 (53.928–227.88)
Pelagic	1970–1989	31.656 (21.656–53.025)	21.235 (14.26–41.864)	288.338 (172.435–691.15)
	1990–2006	11.331 (7.587–27.397)	3.8 (2.45–11.228)	5.527 (3.002–22.122)

Mode and 5% and 95% quantiles of the posterior distribution from 100,000 samples.

**Table 5:** Probability of niches for double-crested cormorant and pelagic cormorant in the two time periods having different centroid locations and distance between centroids in 2, 3, and 5 dimensions.

	dim	p	distance
Double-crested	2D	0.6	2.19
	3D	0.558	2.28
	5D	0.571	2.49
Pelagic	2D	0.208	0.875
	3D	0.207	0.881
	5D	0.131	0.543

In both species, niche overlap between the two periods was lower in higher dimensions, as expected. As niche volume decreased, the overlap of the late period on the early period overlapped little in double-crested cormorants in all dimensional approaches (see Table 6 for directional overlap details). However, the early period niche showed a high overlap with the later period niche in 2D (96.99%) but became minimal toward higher dimensions (11.6% in 5D). In contrast, pelagic cormorants’ later period niche overlapped more than 50% on the early period niche throughout all dimensional approaches (Table 6). Considering the niche size changes from lower to higher dimensional approaches (Table 4) these changes in overlap show differences in niches when incorporating new dimensions.

**Table 6:** Percentage of directional niche overlap between periods for double-crested cormorant and pelagic cormorant (periods in rows overlapping onto periods in columns) for 2D, 3D, and 5D approaches.

	2d		3d		5d	
	1970–1989	1990–2006	1970–1989	1990–2006	1970–1989	1990–2006
Double-crested						
1970–1989	NA	19.28	NA	20.27	NA	8.38
1990–2006	**96.99**	NA	47.22	NA	11.6	NA
Pelagic						
1970–1989	NA	**53.08**	NA	29.42	NA	2
1990–2006	**91.68**	NA	**88.78**	NA	**56**	NA

Bolded are those pairs with high probability values (> 50%). Overlap direction is period 1 (row header) with period 2 (column header).

## Discussion

The use of higher dimensional approaches to assess niche size, overlaps, and community metrics, improved our capacity to detect differences and pattern changes in a community assemblage of seabirds, as suggested by Bowes et al.^40^. Specifically, the isospace produced considering a five-dimension “Hutchinson” hypervolume, made possible via the use of sulphur and amino acid-specific isotopes, improved our understanding of niche space compared to the use of bulk carbon and nitrogen. Moreover, PC1 of nitrogen was associated with baseline δ^15^N levels and PC2 was associated with trophic position, illustrating that using bulk δ^15^N as a metric of trophic position could lead to incorrect inferences. Polito et al.^80^ also found that two species of penguin were differentiated when using essential amino acids of carbon isotope values, which did not occur when using only bulk isotopic values. Ranking of niche size and Layman metrics (centroid distance, nearest neighbour distance, and standard deviation of the nearest neighbour distance) were remarkably similar across dimensions, although absolute values were larger in higher dimensions, as suggested by Mammola^81^. Thus, the overall topology of the guilds’ n-dimensional space did not change, but species segregated differently from one another in higher dimensions. As herring stocks decreased over time since the 1950s due to depletion by commercial fisheries^82^, a generalist species’ (double-crested cormorant) hypervolume remained constant while a specialist species’ (pelagic cormorant) hypervolume decreased, illustrating how the topology can change over time.

The increase in dimensionality enlarged the niche size for each species and showed a shift in the trend in size in 5D. We observed a consistent pattern of niche size, with double-crested cormorants having the largest niche of all five species, followed by the rhinoceros auklet, and pelagic cormorant. In 2D and 3D, ancient murrelet and Leach’s storm-petrel followed with the smallest niches, the latter showing the smallest niche. But in 5D these two species switched positions, as murrelets were the species with the smallest niche. Thus, Leach’s storm-petrel had a small dietary niche along the classic isotopic dimensions (trophic position, offshore/nearshore habitat), but extended that niche through finer scale dimensions revealed by amino acid-specific isotopes, which is consistent with the large habitat size (large foraging range over offshore habitats) and variable diet (myctophid fish and invertebrates) in that species. Niche size calculation in higher dimensions seems to be affected somewhat by the formula incorporated by Swanson et al.^45^, and should be interpreted with caution for analyses with more than three dimensions.

Increasing dimensions significantly affected overlap among species (Fig. 2 and Table S7) from lower to higher dimensional approaches. Although a pattern can be observed of less overlap when using more dimensions compared to fewer dimensions, some species pairs do not follow the expected pattern. Some species-pairs slightly increased in overlap with the increase from 2 to 3D (auklets-murrelets, murrelets-double-crested, murrelets-pelagic, etc.), but then in most cases, overlap between pairs dropped dramatically in 5D. That change in overlap can be associated with the changes in the niche size of each species or group. When increasing dimensions, larger niche species tend to increase niche volume, but that increase moves away from the corresponding species pair, whereas small niche species retained the overlapping section of their niche in the same proportion as to the larger niche species. That result would be consistent with the described difference in the diet of, for example, the two cormorants where both are eating similar fish species, but pelagic cormorants feed at lower trophic levels and have a more restricted diet than double-crested cormorants^36,58^.

Conversely, increasing information by incorporating additional dimensions into niche size calculations can produce changes in the rankings of previously observed niche sizes. Specifically, smaller niche species can experience an increase in niche size in higher dimensional approaches, and surpass other species compared to lower dimensional approaches. That was seen in our analyses in storm-petrels vs. murrelets in 5D, presumably associated with their differences in prey^83,84^. In addition, contrary to what would be expected, the overlap of certain species, such as murrelets, at higher dimensions remains somewhat important with the other species, like fish-eating species, pelagic and double-crested cormorants (Table S7).

### Layman metrics in a multidimensional space

All Layman dispersion metrics calculated increased, in different magnitudes, from lower to higher dimensional approaches, as expected. Species packing increased only slightly from low to higher dimensions, showing a similar structure of the assemblage in the community. Conversely, the greater increase in density of species packing (NND) and evenness of species packing (SDNND) with higher dimensions demonstrates that the use of new dimensions incorporates new information about all species. That may allow for better comparison and detection of changes that would otherwise be unnoticed with the use of fewer dimensions or only bulk isotopes of carbon and nitrogen as suggested by Bowes et al.^40^.

### Changes in time for cormorants

Cormorants show a very distinct temporal niche trend, especially in 5D^42^. A lower dimensional approach shows an important difference in niche size change in time for double-crested and, of lower magnitude, for pelagic cormorants, which becomes switched in higher dimensions (Table 4). The information incorporated by the higher resolution of amino acid-specific isotopes shows a significant reduction in the isotopic niche of pelagic cormorants, which does not occur in double-crested cormorants. Although niche sizes change, the overlap of pelagic cormorant niches over time is greater, whereas double-crested niche overlap in time is minimal in higher dimensions. There is some evidence that both double-crested and pelagic cormorant populations in Pacific Canada have declined in recent decades, likely due to several factors, including prey availability^57^. The once enormous herring spawns in the Salish Sea^85^, occurring during the pre-laying period for cormorants, are greatly reduced in size and the reduction of this prey may be the reason for the changes. Indeed, the changes over time are largely in carbon axes rather than trophic position (PC2 of δ^15^N), which is consistent with a change from schooling to benthic prey. The niche size stability in higher dimensions in double-crested cormorants during the last decades may reflect some flexibility in the capacity of changing prey types but retain a similar niche breadth, by switching to benthic or freshwater prey. Contrarily, the niche size reduction seen in pelagic cormorants, but greater overlap in the later period, may reflect changes in fish abundance or diversity near the coast and rocky bottoms, which may mean a lesser capacity to shift prey type.

Our research demonstrates how the use of higher n-dimensional approaches, as suggested by Hutchinson^1^, can incorporate greater details and show better segregation patterns in a community, especially with the combinations of compound-specific amino acid isotopes. Those additional isotopes can provide valuable, less biased information on the ecological roles of species within a community, and at the same time overcome the lack of information contained only in two niche proxies. The overall topology of the community remained constant, but patterns of overlap and segregation among species varied significantly with increased dimensions, as well as changes in specific niche hypervolume size. We encourage researchers to incorporate more dimensions (sulphur, amino acids) into isotopic niche models to detect more accurate differences in niche composition.

### Supplementary Information

Supplementary Information 1.

Supplementary Information 2.

## Data Availability

Original raw data included as supplementary materials. R code publicly available at https://github.com/francisvolh/multiDimNiche.
