# Supplementary material for: Amino acid-specific isotopes reveal changing five-dimensional niche segregation in Pacific seabirds over 50 years
Source: Sci Rep. 2024 Apr 3;14:7899. doi: 10.1038/s41598-024-57339-w (PMC10991557; doi:10.1038/s41598-024-57339-w)
Supplement: Supplementary file 2 — Supplementary Information 2. [file 41598_2024_57339_MOESM2_ESM.docx]

**Supplementary Materials**


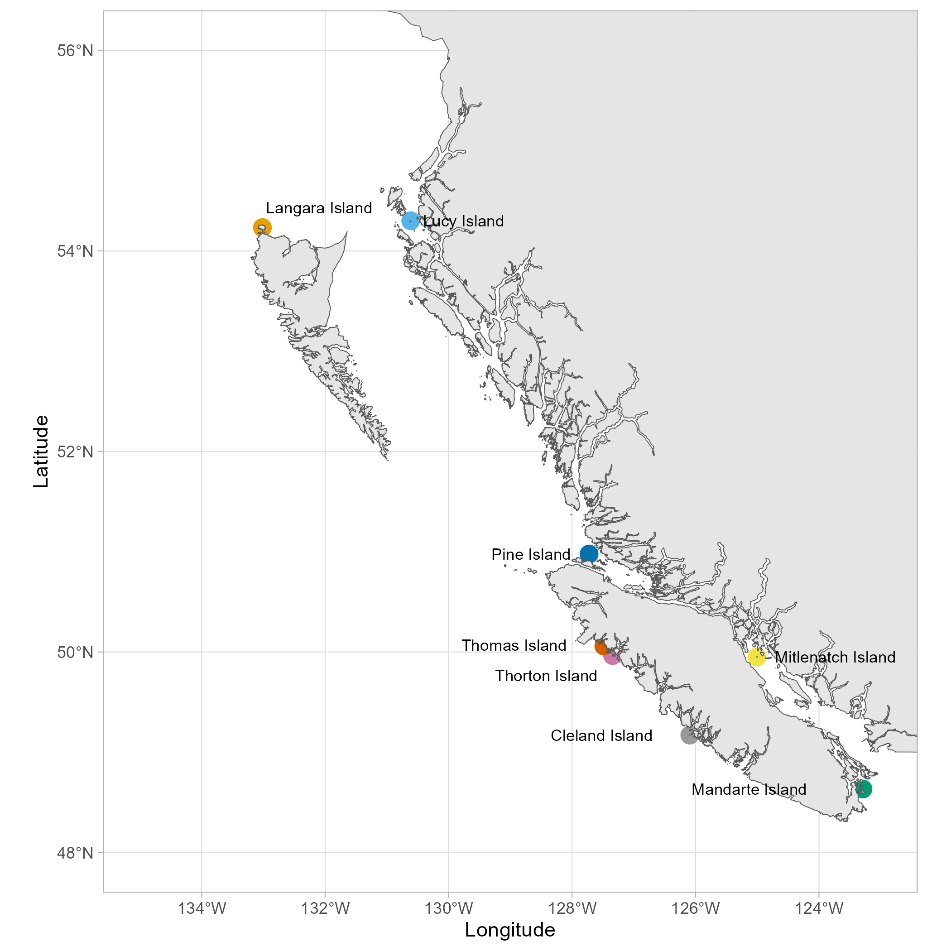


Figure S1. Sampling locations for 6 seabird species along the British Columbia coast. Species per location: ANMU = Langara Is.; DCCO = Mandarte Is., PECO = Mandarte Is., Mitlenatch Is.; LSPE = Cleland Is., Thomas Is., Thorton Is.; RHAU = Lucy Is., Pine Is.)

Table S1. Study species, abbreviation codes, and samples sizes in each sampling period.

| Species | Code | 1970-1989 | 1990-2006 |
| --- | --- | --- | --- |
| Ancient murrelet *Synthliboramphus antiquus* | ANMU | 2 | 4 |
| double-crested cormorant *Phalacrocorax auritus* | DCCO | 6 | 11 |
| Leach’s Sstorm-petrel Oceanodrama leucorhoa | LSPE | 8 | 3 |
| pelagic cormorant *Phalacrocorax pelagicus* | PECO | 13 | 6 |
| Rhinoceros auklet *Cerorhinca monocerata* | RHAU | 0 | 10 |

Table S2. PCA loadings for all amino acids for carbon and nitrogen variables

|  | PCA.1 | PCA.2 |  |  | PCA.1 | PCA.2 |
| --- | --- | --- | --- | --- | --- | --- |
| dN_Ala | 16.07 | 1.90 |  | dC_Ala | 11.90 | 0.00 |
| dN_Asp | 7.70 | 23.67 |  | dC_Val | 11.87 | 1.61 |
| dN_Glx | 8.45 | 9.15 |  | dC_Gly | 9.12 | 2.85 |
| dN_Gly | 7.20 | 1.16 |  | dC_Ile | 8.65 | 15.04 |
| dN_Ile | 15.76 | 3.42 |  | dC_Leu | 11.90 | 1.50 |
| dN_Leu | 14.96 | 0.01 |  | dC_Pro | 6.16 | 50.18 |
| dN_Lys | 2.56 | 19.72 |  | dC_Asp | 7.63 | 1.58 |
| dN_Phe | 3.63 | 30.57 |  | dC_Phe | 12.32 | 0.60 |
| dN_Pro | 7.36 | 10.07 |  | dC_Glx | 9.70 | 13.23 |
| dN_Val | 16.32 | 0.33 |  | dC_Lys | 10.74 | 13.42 |


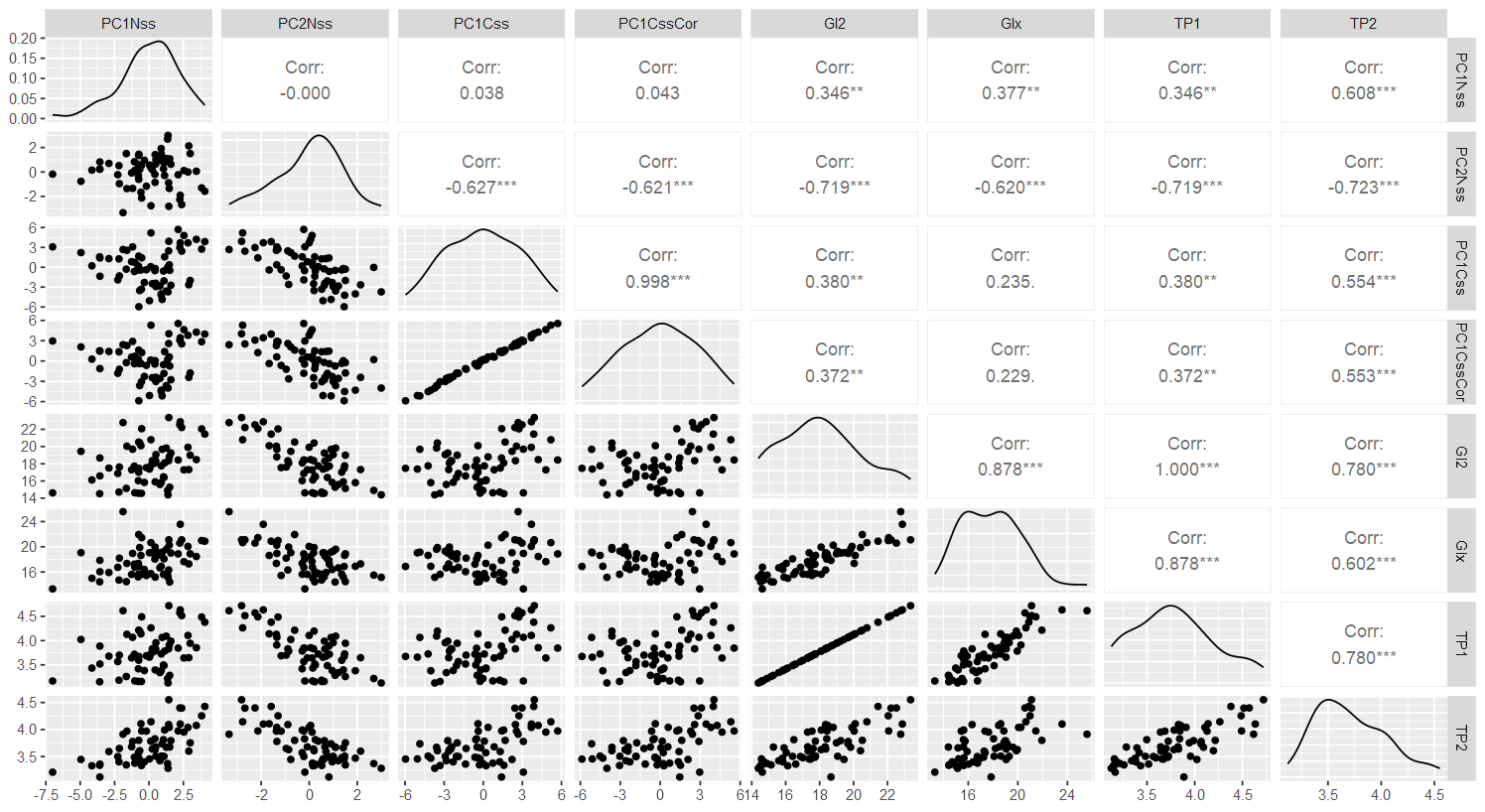


Figure S2. Correlation matrix for PCA components of nitrogen and carbon δ values of amino acids and trophic indices (Glx = Glx-Lys, Gl2 = Glx-Phe, TP1 = trophic position based on Glx, TP2 = trophic position based on 6 trophic amino acids). TP1 and TP2 based on Gagne et al (2018) ^71,72^. PC1CssCor = PC1 of carbon δ values of amino acids after correction for Suess effect

Table S3. Centroid locations for each dimension of the 5-dimensional model of five species of seabirds on the coast of British Columbia. The first two PCA components represent all amino acid specific carbon and nitrogen isotopes, and standardized bulk sulphur. Mean and quantiles (2.5% and 97.5% in brackets) of the posterior distribution from 100 000 samples. For species abbreviations see Table S1.

|  | PC1Css | PC2Css | PC1Nss | PC2Nss | stdDelta.S |
| --- | --- | --- | --- | --- | --- |
| ANMU | 1.69  (0.92-2.47) | -0.47  (-1.22-0.28) | -4  (-5.67--2.33) | -0.06  (-0.54-0.42) | 0.2  (0.1-0.3) |
| DCCO | -2.88  (-3.78--1.98) | 0.14  (-0.13-0.41) | 0.68  (0.02-1.34) | 1.27  (0.88-1.65) | -1.14  (-1.61--0.66) |
| LSPE | 3.82  (3.15-4.49) | -0.36  (-0.89-0.18) | 2.41  (1.71-3.1) | -1.3  (-2.02--0.58) | 1.19  (1.14-1.24) |
| PECO | -0.93  (-1.51--0.35) | -0.45  (-0.68--0.21) | -0.62  (-1.23-0) | 0.28  (-0.07-0.64) | 0.12  (0.04-0.2) |
| RHAU | 1.46  (0.49-2.42) | 1.29  (0.9-1.67) | -0.23  (-1.12-0.66) | -1.22  (-1.99--0.45) | 0.28  (-0.27-0.82) |

Table S4. Bhattacharrya Coefficient for the probability of overlap between posterior distribution of each independent isotopic dimension (first two PCA components for all amino acid specific carbon and nitrogen isotopes and standardized bulk sulphur) for species pairs. Bolded are those with high probability values (>0.8). For species abbreviations see Table S1

| PC1Css |  |  |  |  |
| --- | --- | --- | --- | --- |
|  | DCCO | LSPE | PECO | RHAU |
| ANMU | 0.001 | 0.048 | 0.015 | **0.950** |
| DCCO | 1.000 | 0.000 | 0.064 | 0.001 |
| LSPE | 0.000 | 1.000 | 0.000 | 0.048 |
| PECO | 0.064 | 0.000 | 1.000 | 0.031 |
| RHAU | 0.001 | 0.048 | 0.031 | 1.000 |
|  |  |  |  |  |
| PC2Css |  |  |  |  |
|  | DCCO | LSPE | PECO | RHAU |
| ANMU | 0.424 | 0.**966** | 0.**793** | 0.048 |
| DCCO | 1.000 | 0.449 | 0.093 | 0.016 |
| LSPE | 0.449 | 1.000 | **0.853** | 0.016 |
| PECO | 0.093 | 0.853 | 1.000 | 0.000 |
| RHAU | 0.016 | 0.016 | 0.000 | 1.000 |
|  |  |  |  |  |
| PC1Nss |  |  |  |  |
|  | DCCO | LSPE | PECO | RHAU |
| ANMU | 0.018 | 0.002 | 0.058 | 0.059 |
| DCCO | 1.000 | 0.068 | 0.156 | 0.501 |
| LSPE | 0.068 | 1.000 | 0.000 | 0.017 |
| PECO | 0.156 | 0.000 | 1.000 | **0.854** |
| RHAU | 0.501 | 0.017 | **0.854** | 1.000 |
|  |  |  |  |  |
| PC2Nss |  |  |  |  |
|  | DCCO | LSPE | PECO | RHAU |
| ANMU | 0.040 | 0.172 | 0.697 | 0.225 |
| DCCO | 1.000 | 0.005 | 0.054 | 0.004 |
| LSPE | 0.005 | 1.000 | 0.047 | **0.993** |
| PECO | 0.054 | 0.047 | 1.000 | 0.067 |
| RHAU | 0.004 | **0.993** | 0.067 | 1.000 |
|  |  |  |  |  |
| stdDeltaS |  |  |  |  |
|  | DCCO | LSPE | PECO | RHAU |
| ANMU | 0.005 | 0.000 | **0.662** | **0.631** |
| DCCO | 1.000 | 0.000 | 0.006 | 0.049 |
| LSPE | 0.000 | 1.000 | 0.000 | 0.045 |
| PECO | 0.006 | 0.000 | 1.000 | 0.509 |
| RHAU | 0.049 | 0.045 | 0.509 | 1.000 |

Table S5. Probability that the two species have different centroid location values in 2, 3, and 5 dimensions (A, B, C respectively) and distance between centroid locations between species pairs in 2, 3, and 5 dimensions (D, E, F respectively). For species abbreviations see Table S1.

| A) | ANMU | DCCO | LSPE | PECO | RHAU |
| --- | --- | --- | --- | --- | --- |
| ANMU | 0 | 0.998 | 0.999 | 0.974 | 0.929 |
| DCCO | 0.998 | 0 | 1 | 0.935 | 0.995 |
| LSPE | 0.999 | 1 | 0 | 1 | 0.991 |
| PECO | 0.974 | 0.935 | 1 | 0 | 0.925 |
| RHAU | 0.929 | 0.995 | 0.991 | 0.925 | 0 |
|  |  |  |  |  |  |
| B) | ANMU | DCCO | LSPE | PECO | RHAU |
| ANMU | 0 | 0.997 | 0.999 | 0.972 | 0.922 |
| DCCO | 0.997 | 0 | 1 | 0.96 | 0.996 |
| LSPE | 0.999 | 1 | 0 | 1 | 0.994 |
| PECO | 0.972 | 0.96 | 1 | 0 | 0.917 |
| RHAU | 0.922 | 0.996 | 0.994 | 0.917 | 0 |
|  |  |  |  |  |  |
| C) | ANMU | DCCO | LSPE | PECO | RHAU |
| ANMU | 0 | 0.998 | 0.998 | 0.969 | 0.953 |
| DCCO | 0.998 | 0 | 1 | 0.973 | 0.998 |
| LSPE | 0.998 | 1 | 0 | 1 | 0.994 |
| PECO | 0.969 | 0.973 | 1 | 0 | 0.982 |
| RHAU | 0.953 | 0.998 | 0.994 | 0.982 | 0 |
|  |  |  |  |  |  |
| D) | ANMU | DCCO | LSPE | PECO | RHAU |
| ANMU | 0 | 6.58 | 6.77 | 4.31 | 3.82 |
| DCCO | 6.58 | 0 | **6.95** | **2.39** | 4.47 |
| LSPE | 6.77 | 6.95 | 0 | 5.65 | 3.6 |
| PECO | 4.31 | 2.39 | 5.65 | 0 | 2.47 |
| RHAU | 3.82 | 4.47 | 3.6 | 2.47 | 0 |
|  |  |  |  |  |  |
| E) | ANMU | DCCO | LSPE | PECO | RHAU |
| ANMU | 0 | 6.71 | 6.84 | 4.3 | 3.82 |
| DCCO | 6.71 | 0 | **7.33** | 2.7 | 4.71 |
| LSPE | 6.84 | 7.33 | 0 | 5.76 | 3.72 |
| PECO | 4.3 | 2.7 | 5.76 | 0 | **2.49** |
| RHAU | 3.82 | 4.71 | 3.72 | 2.49 | 0 |
|  |  |  |  |  |  |
| F) | ANMU | DCCO | LSPE | PECO | RHAU |
| ANMU | 0 | 6.89 | 6.98 | 4.35 | 4.41 |
| DCCO | 6.89 | 0 | **7.8** | **2.96** | 5.47 |
| LSPE | 6.98 | 7.8 | 0 | 5.99 | 4.11 |
| PECO | 4.35 | 2.96 | 5.99 | 0 | 3.42 |
| RHAU | 4.41 | 5.47 | 4.11 | 3.42 | 0 |


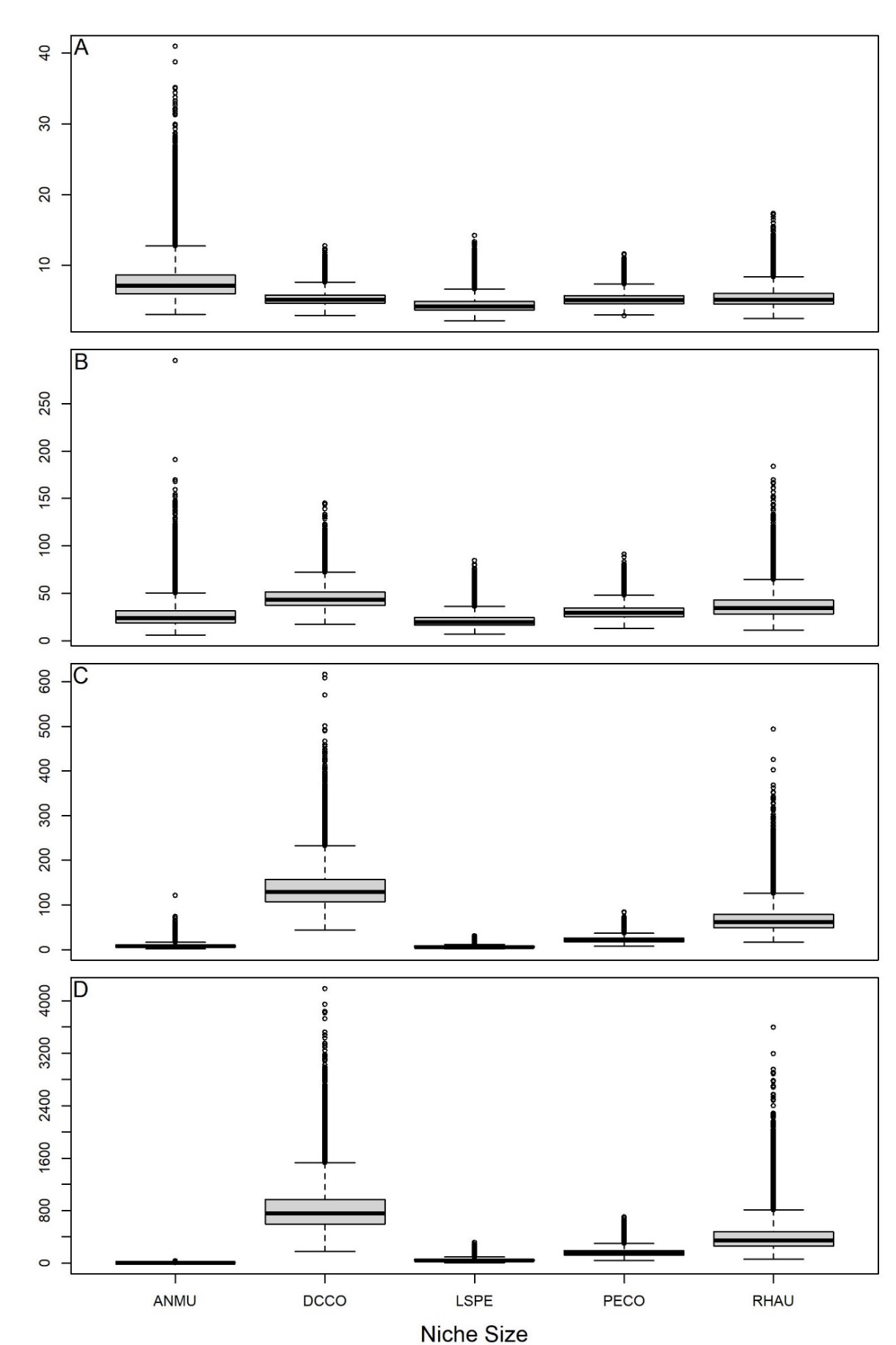


Figure S2 Niche sizes for five species of seabirds on the coast of British Columbia from 100 000 samples for 2D, 3D, and 5D approaches (A), B) and C) respectively). For species abbreviations see Table S1

Table S6. Bhattacharrya Coefficient for the probability of overlap between posterior distribution of niche sizes for species pairs for each dimensional approach (A = 2D, B = 3D, C = 5D). Bolded are those pairs with high probability values (>0.8). For species abbreviations see Table S1.

| A) | DCCO | LSPE | PECO | RHAU |
| --- | --- | --- | --- | --- |
| ANMU | 0.60 | **0.95** | **0.87** | **0.85** |
| DCCO | 1 | 0.34 | 0.70 | **0.89** |
| LSPE | 0.34 | 1 | 0.73 | 0.64 |
| PECO | 0.70 | 0.73 | 1 | **0.94** |
| RHAU | **0.89** | 0.64 | **0.94** | 1 |
|  |  |  |  |  |
| B) | DCCO | LSPE | PECO | RHAU |
| ANMU | 0.00 | **0.95** | 0.35 | 0.05 |
| DCCO | 1 | 0.00 | 0.00 | 0.53 |
| LSPE | 0.00 | 1 | 0.13 | 0.00 |
| PECO | 0.00 | 0.13 | 1 | 0.23 |
| RHAU | 0.53 | 0.00 | 0.23 | 1 |
|  |  |  |  |  |
| C) | DCCO | LSPE | PECO | RHAU |
| ANMU | 0.00 | 0.02 | 0.00 | 0.00 |
| DCCO | 1 | 0.00 | 0.08 | 0.63 |
| LSPE | 0.00 | 1 | 0.25 | 0.06 |
| PECO | 0.08 | 0.25 | 1 | 0.57 |
| RHAU | 0.63 | 0.06 | 0.57 | 1 |

Table S7. Percentage of niche overlap between all species (Species A rows overlapping onto Species B in columns) for 2D, 3D, and 5D approaches (A), B) and C) respectively). Bolded are those pairs with high probability values (>50%), except in 5D where the highest value is bolded, but below 50%. For species abbreviations see Table S1.

| A) | ANMU | DCCO | LSPE | PECO | RHAU |
| --- | --- | --- | --- | --- | --- |
| ANMU | NA | 9.45 | 0.5 | 25.78 | 36.09 |
| DCCO | 5.42 | NA | 0.62 | **56.95** | 26.84 |
| LSPE | 0.96 | 1.57 | NA | 0.81 | 31.32 |
| PECO | 23.33 | **79.66** | 0.63 | NA | **63.78** |
| RHAU | 33.87 | 39.74 | 14.01 | **53.14** | NA |
|  |  |  |  |  |  |
| B) | ANMU | DCCO | LSPE | PECO | RHAU |
| ANMU | NA | 12.84 | 0 | 30 | 13.39 |
| DCCO | 0.83 | NA | 0.07 | 9.88 | 9.07 |
| LSPE | 0 | 2.5 | NA | 0 | 0.22 |
| PECO | 11.32 | **75.51** | 0 | NA | 32.83 |
| RHAU | 4.08 | 32.42 | 0.02 | 17.5 | NA |
|  |  |  |  |  |  |
| C) | ANMU | DCCO | LSPE | PECO | RHAU |
| ANMU | NA | 7.43 | 0 | 24.46 | 0.36 |
| CO | 0.02 | NA | 0 | 5.71 | 0.59 |
| LSPE | 0 | 0.03 | NA | 0 | 0.02 |
| PECO | 0.43 | ***45.86*** | 0 | NA | 0.35 |
| RHAU | 0 | 1.44 | 0 | 0.24 | NA |


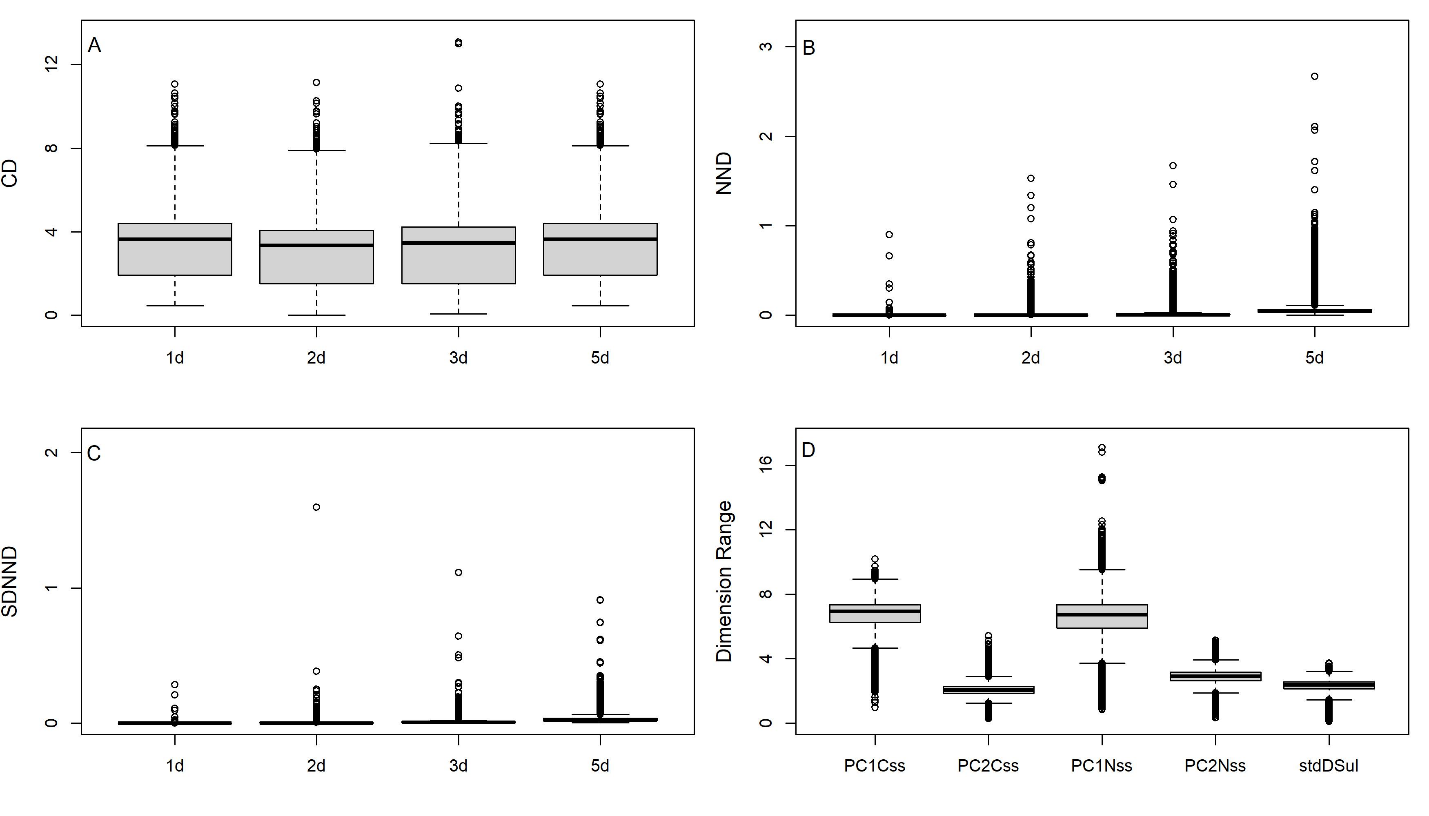


Figure S3. A) Centroid Distance (CD), B) Nearest-Neighbor-Distance (NND), C) Standard deviation of the nearest neighbor distance (SDNND), and D) range of each dimension for all five species of seabirds in 1D, 2D, 3D, and 5D.

Table S8. Centroid locations for each dimension for the 5-dimensional model double-crested cormorant (DCCO) and pelagic cormorant (PECO) in the British Columbia coast. First two PCA components for all amino acid specific carbon and nitrogen isotopes and standardized bulk sulphur. Mean and 2.5% and 97.5% quantiles (in brackets) of the posterior distribution from 100 000 samples.

| DCCO | PC1Css | PC2Css | PC1Nss | PC2Nss | stdDelta.S |  |
| --- | --- | --- | --- | --- | --- | --- |
| 1970-1989 | -1.59 (-3.98,0.78) | 0.59 (0.11,1.08) | 0.67 (-1.44,2.77) | 1.65 (0.9,2.4) | -0.79 (-1.78,0.2) | |
| 1990-2006 | -3.59 (-4.2,-2.99) | -0.11 (-0.49,0.27) | 0.69 (0.22,1.15) | 1.06 (0.5,1.62) | -1.33 (-1.98,-0.68) | |
| PECO | PC1Css | PC2Css | PC1Nss | PC2Nss | stdDelta.S | |
| 1970-1989 | -0.99 (-1.76,-0.23) | -0.58 (-0.94,-0.22) | -0.79 (-1.69,0.11) | 0.35 (-0.17,0.87) | 0.15 (-0.05,0.34) | |
| 1990-2006 | -0.79 (-2.09,0.51) | -0.15 (-0.62,0.31) | -0.24 (-1.09,0.61) | 0.14 (-0.57,0.85) | 0.06 (-0.37,0.49) | |

Table S9. Bhattacharrya Coefficient for the probability of overlap between the posterior distribution of each independent isotopic dimension (first two PCA components for all amino acid specific carbon and nitrogen isotopes and standardized bulk sulphur) between time periods for double-crested cormorant (DCCO) and pelagic cormorant (PECO). Bolded are those with high probability values (>0.8)

|  | PC1Css | PC2Css | PC1Nss | PC2Nss | stdDelta.S |
| --- | --- | --- | --- | --- | --- |
| DCCO | 0.280671 | 0.085839 | 0.690387 | 0.5351465 | *0.7229053* |
| PECO | **0.937017** | 0.568858 | **0.8109186** | **0.9207483** | **0.8706205** |

Table S10. Bhattacharrya coefficient of niche size distributions of PECO and DCCO in different time intervals

|  | 2d | 3d | 5d |
| --- | --- | --- | --- |
| PECO | 0.4093703 | 0.1654886 | 0.003084003 |
| DCCO | 0.006725132 | 0.4647408 | **0.9800721** |

Table S11. Layman metrics for PECO and DCCO

| PECO | 5D | 3D | 2D |
| --- | --- | --- | --- |
| CD | 0.8 (0.3-1.77) | 0.59 (0.13-1.38) | 0.58 (0.11-1.38) |
| NND | 0.05 (0.02-0.16) | 0.01 (0-0.03) | 0.01 (0-0.01) |
| SDNND | 0.04 (0.01-0.1) | 0.01 (0-0.03) | 0 (0-0.01) |
| Range PC1Css | 1.7 (0.79-3.23) | 1.48 (0.72-2.71) | 1.48 (0.72-2.68) |
| Range PC2Css | 0.9 (0.52-1.41) |  |  |
| Range PC1Nss | 1.59 (0.85-2.66) | 1.44 (0.78-2.31) | 1.44 (0.78-2.32) |
| Range PC2Nss | 1.03 (0.51-1.86) |  |  |
| Range stdDelta.S | 0.54 (0.24-1.05) | 0.21 (0.12-0.35) |  |

| DCCO | 5D | 3D | 2D |
| --- | --- | --- | --- |
| CD | 1.53 (0.62-3.61) | 1.33 (0.4-3.16) | 1.26 (0.35-3.04) |
| NND | 0.08 (0.03-0.25) | 0.03 (0-0.07) | 0.04 (0-0.02) |
| SDNND | 0.06 (0.02-0.16) | 0.02 (0-0.06) | 0.01 (0-0.03) |
| Range PC1Css | 3.76 (2.08-6.33) | 3.52 (2.06-5.59) | 3.53 (2.04-5.67) |
| Range PC2Css | 1.18 (0.78-1.73) |  |  |
| Range PC1Nss | 2.41 (0.8-5.07) | 2.13 (0.72-4.37) | 2.12 (0.71-4.36) |
| Range PC2Nss | 1.34 (0.75-2.18) |  |  |
| Range stdDelta.S | 1.53 (0.79-2.66) | 1.36 (0.74-2.23) |  |

Table S12. Ranges of posterior distributions of all independent isotopic dimensions, with 100000 iterations.

|  |  | Ranges |
| --- | --- | --- |
|  | PC1Css | 6.7(4.83-7.95) |
|  | PC2Css | 2.02(0.94-2.65) |
|  | PC1Nss | 6.5(3.35-8.75) |
|  | PC2Nss | 2.92(1.67-3.64) |
|  | stdDelta.S | 2.23(1.12-2.88) |
